# Interruptions During Sign-out Between Emergency Medicine Residents Before and After Implementation of Group Sign-out Process

**DOI:** 10.5811/westjem.59486

**Published:** 2023-12-22

**Authors:** Andrea Lin, Makenna Brezitski, Marko Zegarac, Sue Boehmer, Robert P. Olympia

**Affiliations:** * Penn State College of Medicine, Hershey, Pennsylvania; † Penn State Hershey Medical Center, Department of Emergency Medicine, Hershey, Pennsylvania; ‡ Division of Biostatistics, Department of Public Health Services, Penn State Hershey Medical Center, Pennsylvania; § Penn State Hershey Children’s Hospital, Department of Pediatrics, Hershey, Pennsylvania

## Abstract

**Introduction:**

Interruptions that occur during sign-out in the emergency department (ED) may affect workflow, quality of care, patient safety, errors in documentation, and resident education. Our objective in this study was to determine the frequency and classification (emergent vs non-emergent, in-person vs phone call) of interruptions that occur during emergency medicine (EM) resident sign-out before and after the institution of a group sign-out process involving residents and attending physicians.

**Methods:**

A convenience sample of sign-out observations between EM residents were observed and coded between April–December 2021. We excluded sign-out observations of pediatric patients (<18 years of age) and observations not conducted in the main ED. Collected data included number of patients signed out during each observation; total duration in minutes for each observation; total number of interruptions during each observation; and type of interruption (emergent vs non-emergent, in-person vs phone call). We further stratified data before and after the institution of a group sign-out process (July 2021).

**Results:**

We performed data analysis on 58 individual and 65 group sign-out observations, respectively. Although the total number of patients signed out, the total duration of sign-outs observed, mean number of patients signed out per minute, and mean duration of sign-out per observation were more for the group sign-out aggregate compared with the individual sign-out aggregate, the total number of interruptions (44 vs 73, *P* = 0.007), number of interruptions per minute (0.05 vs 0.16, *P* < 0.001), total number of non-emergent interruptions (38 vs. 67, P = 0.005), and total number of in-person interruptions (14 vs 44, *P* < 0.001) was less in the group sign-out compared with the individual sign-out totals.

**Conclusion:**

Based on our sample, although the total duration of group sign-out with both residents and an attending was longer than individual resident-to-resident sign-out, the total number of interruptions, number of interruptions per minute, total number of non-emergent interruptions, and total number of in-person interruptions was less in the group sign-out. Group sign-out may be an option to limit the negative effects of interruptions in the ED.

Population Health Research Capsule What do we know about this issue?

*Depending on setting, some signout methods are more effective. No known study has examined quantitative differences between one-to-one versus a group signout at an academic institution.*

What was the research question?

*How do individual versus group signouts in the ED differ in types and frequency of interruptions experienced?*

What was the major finding of the study?

*Group sign-outs take longer (8.0 vs. 13.7 minutes) but experience less frequent interruptions (0.05 vs. 0.16 per minute) when compared to individual sign-outs in the emergency department.*

How does this improve population health?

*Less frequent interruptions during shift change allow for accurate and timely exchange of patient information critical to care during patients’ stays in the emergency department.*

## INTRODUCTION

The sign-out of patient information from one emergency physician to the next is a critical time that requires their undivided attention. Essential information—such as the history of present illness, past medical history, physical examination findings and vital signs, differential diagnosis, emergency department (ED) management, and disposition based on the response to interventions and diagnostic testing—is often communicated during the sign-out process.^1^ Yet the sign-out process is frequently interrupted for both emergent and non-emergent reasons.

Previous literature has described disturbances during the ED shift with clear delineations between “multitasking” and interruptions.^2^ Some authors have also detailed the quantity and quality of interruptions experienced by emergency physicians.^3^ Specifically, interruptions took place most frequently during information exchange at nursing and doctor stations, and in-person interruptions were the most common type of interruption. Such interruptions have resulted in residents experiencing decreased efficiency and productivity ultimately measured by increased documenting time, more frequent patient readmissions, less face-to-face patient interaction time, and additional phone calls from consultants, nurses, and other healthcare staff about incorrect orders.^4^

These interruptions can manifest as loss of critical patient information or misinterpretation of patient status, which ultimately pose a risk to patient safety.^1^ Lastly, oncoming physicians who assume responsibility of patients must summarize each patient encounter and create an addendum in the electronic health record (EHR). As reliance on the EHR increases, errors in documentation and communication may increase due to frequent interruptions during the sign-out process. Unanticipated or anticipated stressors may contribute to mistakes, which may result in auditing of notes or other legal implications that could have been otherwise avoided.^5^ In addition to the effect that interruptions have on the quality of care provided, patient safety, and physician documentation, resident physicians may be subjected to disjointed education when lessons are frequently incongruent, as their peers and educators are often called away to answer questions, address complaints, and assist patients because of interruptions that may or may not be emergent or urgent.^6^

To our knowledge, there have been no published studies examining the frequency and classification of interruptions that occur during an emergency medicine (EM) resident sign-out, nor have there been descriptions of these interruptions based on individual resident to resident vs group sign-out process involving both residents and attending physicians. In this study our objective was to determine the frequency and classification (emergent vs non-emergent, in-person vs phone call) of interruptions that occur during EM resident sign-out at Penn State Hershey Medical Center before and after the institution of a group sign-out process.

## METHODS

We conducted a prospective, observational cohort study at Penn State Hershey Medical Center Level I trauma, a designated, tertiary care academic ED in central Pennsylvania, between April 2021–December 2021. A convenience sample of sign-out observations between EM residents were observed and coded by research assistants (RA). The RAs were instructed to record the time at shift change when residents began to verbally sign out with one another, and document total time as well as frequency and type of interruptions. We excluded sign-out observations of pediatric patients (<18 years of age), sign-out observations involving non-EM residents and medical students, and sign-out observations that were not conducted in the attending physician/nurses’ station of the main ED.

We excluded pediatric patients since the number of interruptions involving medical command and medical/traumatic resuscitations involving children would likely be less frequent. Sign-out observations involving non-EM residents and medical students were also excluded since most emergent interruptions would be handled by EM residents, and the majority of adult patients in the Level I ED were managed by EM residents. We excluded sign-outs that occurred outside the attending physician/nurses’ station of the main ED (only applicable during the individual sign-out phase) since we wanted to capture all in-person interruptions due to the nature of their central location.

Emergency medicine residents were notified by email regarding the purpose of the research study, the role of the RA during the sign-out process, and that the presence of the RA would not affect the overall process of sign-out. Nurses and other staff were not aware this study was ongoing. Due to the nature of the teaching hospital, the presence of two additional medical students in an area with high visibility and other learners would be unlikely to influence the frequency of interruptions.

Data collected by the RA for each sign-out observation included the following: number of patients signed out during each observation; total duration in minutes for each observation; total number of interruptions during each observation; and type of interruption (emergent vs non-emergent, in-person vs phone call). Emergent interruptions included command calls (requests) or medical/traumatic resuscitations for patients in the ED, and often occurred by phone call or overhead announcements. Non-emergent interruptions included in-person and phone call interruptions for scenarios that did not require the resident’s immediate response or action (non-resuscitation scenarios). The RAs were available to observe and collect data during the three sign- out periods spread out during the day: 6 am to 8 am, 2 pm to 6 pm, and 10 pm to midnight. The total time of sign-out included whether the resident had to address either emergent or non-emergent interruptions and the sign-out would resume where it had been left off following resolution of the interruptions.

**Figure 1.**
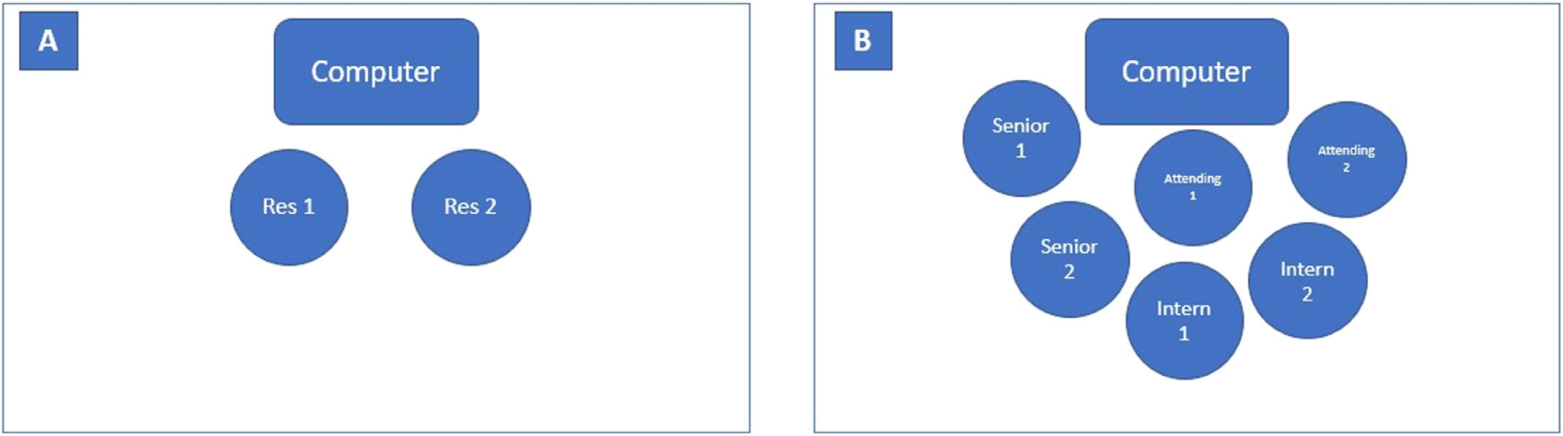
Diagram of individual sign-out (A) compared to group sign-out (B). Individual sign-out consisted of the outgoing resident (Res 1) signing out patients to the incoming resident (Res 2); this would be intern to intern, senior to senior, etc. The group sign-out process included the outgoing attending, senior, and intern (1) signing out patients to the incoming attending, senior, and intern (2). With this group sign-out format, each member of the physician team heard about all patients. *Res*, resident.

At our institution we adopted a group sign-out process involving all residents and one attending physician starting on July 1, 2021, with the hope of increasing attending physician presence at sign-out to streamline care of patients and limit errors associated with the sign-out process, as well as increase the amount of education provided to all learners. Prior to July 1, 2021, the sign-out process occurred “one-on-one” between the outgoing and incoming resident around computers located at the attending physician/nurses’ station of the main ED. After July 1, 2021, the sign-out process occurred as a group of attending physicians, residents and medical students, discussing all the patients assigned to the team, around the attending computer located at the attending physician/nurses’ station of the main emergency department (see Figure 1). Each EM resident carries a mobile phone assigned to them for each shift, and can be contacted at any time during their shift by ED or other hospital staff (such as consultants, primary care physicians, social workers, patient logistics, etc).

Data collected by the RA were imported in REDcap, a secure, web-based software platform designed to support data capture for research studies hosted at Penn State. Descriptive statistics were generated and included the means, medians, standard deviations and 95% confidence intervals for continuous variables; percentages were calculated for categorical variables. To compare the group vs individual sign-out process as continuous variables, we used a two-sample *t*-test. To compare between the group and individual sign-out process as categorical variables, we used a binomial test for proportions. All tests were two-sided and were considered statistically significant if the *P*-value was <0.05. The statistical analysis was performed using SAS software, version 9.4 (SAS Institute Inc, Cary, NC).

The institutional review board approved the study. No funding or grants were received for research or preparation of this manuscript.

## RESULTS

We performed data analysis on 58 individual sign-out observations and 65 group sign out observations. (Six individual sign-out observations and one group sign-out observation was excluded due to missing data on duration of sign-out observation). Although the total number of patients signed out, the total duration of sign- out observed, mean number of patients signed out per minute, and mean duration of sign-out per observation were more for the group sign-out aggregate compared with the individual sign-out aggregate, the total number of interruptions (44 vs 73, *P* = 0.007), number of interruptions per minute (0.05 vs 0.06, *P* < 0.001), total number of non-emergent interruptions (38 vs 67, 0.005), and total number of in-person interruptions (14 vs 44, *P* < 0.001) was less in the group sign-out aggregate compared with the individual sign-out aggregate (Table 1).

**Table 1.** Characteristics of individual and group sign-out observations.

	Individual sign outs, mean ± SD (95% CI)	Group sign outs, mean ± SD (95% CI)	*P*-value
Mean number of patients signed out per minute	0.63 ± 0.26 (0.56, 0.70)	0.79 ± 0.26 (0.73, 0.86)	<0.001
Mean duration of sign-out per observation (minutes)	7.97 ± 5.79 (6.44, 9.49)	13.66 ± 5.34 (12.34, 14.99)	<0.001
Total number of interruptions recorded for all observations	73 (62.4%)	44 (37.6%)	0.007
Mean number of interruptions per minute	0.158 ± 0.21 (0.10, 0.22)	0.049 ± 0.06 (0.03, 0.06)	<0.001
Total number of emergent interruptions	6 (50.0%)^a^	6 (50.0%)^b^	1.00
Total number of command calls	4 (50.0%)	4 (50.0%)	1.00
Total number of non-emergent interruptions	67 (63.8%)	38 (36.2%)	0.005
Total number of phone call interruptions	23 (46.9%)	26 (53.1%)	0.67
Total number of in-person interruptions	44 (75.9%)	14 (24.1%)^c^	<0.001

a For emergency interruptions during the individual sign-out observation period (n = 6), all interruptions were overhead announcements.

b For emergent interruptions during the group sign-out observation period (n = 6), two interruptions were in person and four interruptions were overhead announcements.

c Of the 14 in-person interruptions during the group sign-out observation period, two were emergent interruptions.

*CI*, confidence interval.

## DISCUSSION

Patient handoff is a critical time that has been estimated to contribute to approximately 80% of medical errors, even outside the ED when transitioning between facilities.^7^ The effectiveness of the hand-off process has been studied in one non-academic institution that uses electronic sign-outs to increase the number of admits from the ED to the inpatient hospital team; however, in doing so, it also increased the total duration of sign-outs.^8^ No prior studies have examined the hand-off process between ED residents to one another, or as a team in the presence of an attending. Yet with the nation’s healthcare systems relying heavily on EHR, electronic sign-outs may be a component of care that could help minimize interruptions and improve efficiency. Thus, understanding the multiple variables that can influence the hand-off process and time can indirectly optimize transitions between patient care. Our study compares the change from one process involving hand-offs between ED resident to ED resident, to a team of residents and one attending at an academic institution. Ultimately, this study provides some insight into the clinical operations that occur during the hand-off process.

The objective of our study was to determine the frequency and classification (emergent vs non-emergent, in-person vs phone call) of interruptions that occur during EM resident sign-out at our institution before and after the institution of a group sign-out process that included multiple residents and one attending physician. Based on our results, although the total duration of group sign-out was longer than individual sign-out, the total number of interruptions, number of interruptions per minute, total number of non-emergent interruptions, and total number of in-person interruptions were less in the group sign-out aggregate. This data signifies that by using a team-based, collective sign-out process, there are fewer interruptions, which could ultimately result in improving ED workflow, quality of care provided, patient safety and outcomes, errors in physician documentation, and resident education provided or learned.

Although there was a statistically significant difference in the total duration of sign-out per observation, the benefits of group sign-out, such as ED attending physician presence to streamline care of patients and limit errors associated with the sign-out process, to increase the amount of education provided to all learners, and to reduce interruptions, may outweigh the increased time spent during the sign-out process. Ultimately this process may be more efficient on multiple fronts, as there were more patients signed out per minute.

The reduction in the total number of interruptions, number of interruptions per minute, total number of non-emergent interruptions, and total number of in-person interruptions after institution of a group sign-out process may be due to the fact that ED staff may be less likely to ask questions and discuss less emergent details when a large team is visibly signing out together, especially when the ED attending physician is present. There was no evidence to suggest that there was a statistically significant difference in the number of emergent interruptions; thus, implementing a group sign-out process would not hinder the team from acting appropriately in the event there was an emergency that appropriately warranted interruption. While the frequency of emergent interruptions cannot be controlled for, the goal of implementing a group sign-out process was to reduce the number of non-emergent interruptions, a decrease that was statistically significant from our study’s data. This can be attributed to multiple reasons that may not be able to be dissected, as a multitude of factors could be at play, including individuals being less inclined to interrupt a large group of individuals, the presence of the attending physician, or greater visibility and awareness of the sign-out process.

## LIMITATIONS

There are several limitations associated with our study. Our study was conducted at a single site at a Level I trauma-designated, tertiary-care academic ED in central Pennsylvania; therefore, our results may not be generalizable to all EDs in the United States. We conducted a convenience sample of sign-out observations limited to adult patients and involving only EM residents, and thus our results may not be applicable to pediatric EDs and sign-out involving non-ED residents. Furthermore, our study was conducted during the COVID-19 pandemic, and because of guidelines to protect healthcare workers (use of personal protective equipment, social distancing, etc), the frequency and classification of interruptions may not reflect non-pandemic times. Lastly, although our objective in this study was to determine the quantity and classification of interruptions that occur during EM resident sign-out, we did not determine whether these interruptions ultimately affected ED workflow, quality of care provided, patient safety and outcomes, errors in physician documentation, and resident education provided or learned.

## CONCLUSION

Based on our sample, although the total duration of group sign-out was longer than individual sign-out, the total number of interruptions, number of interruptions per minute, total number of non-emergent interruptions, and total number of in-person interruptions were less in the group sign-out aggregate compared with the individual sign-out aggregate. Group sign-out may be an option to limit the negative effects of interruptions in the ED, such as the effect on workflow, quality of care provided, patient safety and outcomes, errors in physician documentation, and resident education provided or learned.
